# Productive Overcompensation of Alpine Meadows in Response to Yak Grazing in the Eastern Qinghai-Tibet Plateau

**DOI:** 10.3389/fpls.2019.00925

**Published:** 2019-07-12

**Authors:** Tserang-Donko Mipam, Lin-Ling Zhong, Jian-Quan Liu, Georg Miehe, Li-Ming Tian

**Affiliations:** ^1^Key Laboratory for Bio-Resource and Eco-Environment of Ministry of Education, College of Life Sciences, Sichuan University, Chengdu, China; ^2^Institute of Qinghai-Tibetan Plateau, Southwest Minzu University, Chengdu, China; ^3^State Key Laboratory of Grassland Agro-Ecosystems, Institute of Innovation Ecology, School of Life Sciences, Lanzhou University, Lanzhou, China; ^4^Faculty of Geography, Philipps-University of Marburg, Marburg, Germany

**Keywords:** alpine meadow, net primary production, overcompensation, species diversity, yak grazing

## Abstract

Understanding the interaction between large herbivores and pasture production, especially with respect to the grazing optimization hypothesis, is critical for pasture management and generating theoretical and testable predictions. However, the optimization hypothesis remains contradictory in alpine meadows on the Qinghai-Tibet Plateau (QTP). In this study, we tested the grazing optimization hypothesis using four yak-grazing intensities (no grazing, light grazing, moderate grazing and heavy grazing) in alpine meadow habitats from 2015 to 2017. The results indicated that species diversity did not differ significantly among grazing regimes during the experimental period. However, the aboveground net primary production (ANPP) under moderate grazing consistently significantly exceeded that in control enclosures over 3 years, confirming the grazing optimization hypothesis. Levels of overcompensation varied among grazing intensities and years, and grazing-induced plant compensation may only occur in the short term. The enhanced regrowth of *Poaceae* and *Cyperaceae* induced by yak grazing might contribute to the overall level of overcompensation by plant community. Our results strongly support the grazing optimization hypothesis in the context of alpine meadows grazed by yaks, emphasizing the complex interactions between ANPP, herbivores and other ecological factors in alpine meadows on the QTP. These findings provide new insights for the development of an ecological conservation strategy that will help restore this fragile ecosystem and balance the seemingly incompatible requirements of animal husbandry.

## Introduction

The grazing effect of large herbivores on pasture production is of great importance for grassland management and generating theoretical and testable predictions (Borer et al., 2014; Lezama et al., 2014; Charles et al., 2017). Livestock grazing directly affect soil physicochemical properties and the diversity of above- and belowground organisms, thus leading to changes in grassland biodiversity and production (Wang and Wesche, 2016). It has been reported that livestock type, vegetation type and grazing intensity jointly affect the effects of livestock grazing on grassland biodiversity (Tóth et al., 2018; Török et al., 2018). In addition, herbivores were traditionally considered to reduce plant production because they could cause considerable losses of plant biomass (McNaughton, 1989; Leriche et al., 2003). However, theoretical and empirical studies suggested that the primary production of plant individuals and communities may respond neutrally or positively to herbivore grazing under certain circumstances (Milchunas and Lauenroth, 1993; Knapp et al., 2012; Eldridge et al., 2016; Charles et al., 2017). Thus, the effect of herbivore grazing on plant production remains unclear.

The grazing optimization hypothesis states that a moderate level of grazing may induce redistribution of plant hormones that promote cell division and elongate the remaining meristems, thus enhancing leaf growth and tillering (Siddappaji et al., 2015). This hypothesis, which is also referred as herbivore-stimulated overcompensation, suggests that grazing may stimulate plant over-growth and over-reproduction (Agrawal, 2000; Siddappaji et al., 2015) via mechanisms that plants may use to minimize the negative effects of tissue loss (Jaremo et al., 1999; Leriche et al., 2003; Charles et al., 2017). This hypothesis has been supported by studies conducted in diverse pasture ecosystems, including North American salt marshes and mixed prairies (Hik and Jefferies, 1990; Alward and Joern, 1993), African savannas (McNaughton, 1989; Leriche et al., 2003) and Inner Mongolian grasslands (Liu et al., 2011). However, other studies have suggested that the degree of plant overcompensation varies greatly (Olff and Ritchie, 1998; Bakker et al., 2006; Liu et al., 2015; Charles et al., 2017) due to factors including (1) the formation of plant communities and their co-evolution with the level of herbivore pressure historically present in ecosystems (Vermeire et al., 2005; Holdo et al., 2007; Knapp et al., 2012; Augustine and Derner, 2014; Hanke et al., 2014); (2) the species composition of plant communities and the available resources (Anderson et al., 2007; Borer et al., 2014; Lezama et al., 2014; Eldridge et al., 2016); and (3) the herbivore species and grazing intensities (Eldridge et al., 2011; Charles et al., 2017).

The Qinghai-Tibet Plateau (QTP) is the world’s largest and highest plateau, which covers an area of approximately 2.5 million km^2^ in China and hosts the world’s largest pastoral alpine ecosystem (Miehe et al., 2019). Two distinct types of alpine pasture exist in the QTP: alpine steppe in the northern and northwestern plateau, with the dominant species being *Stipa* (Poaceae) species; and alpine meadow in the eastern and southeastern QTP, dominated by *Kobresia* (Cyperaceae) species (particularly *K. pygmaea*; Zhang et al., 2007; Global Carex Group, 2015). Alpine meadow or sedge pasture, sometimes forming a velvety turf, extends from the Qilian Mountains to the Himalayas, covering an area of 450,000 km^2^ (Miehe et al., 2014). These pastures may be unnatural; it is possible that they are the product of human-induced succession of grassland and forests, and have been continuously maintained by grazing since yaks (*Bos grunniens*) were first domesticated and sheep were introduced to the region (Zhang et al., 2005; Miehe et al., 2014). Additionally, approximately 30% of the QTP grasslands have been degraded in spite of remaining somewhat contentious (Harris, 2010; Miehe et al., 2019). An experiment, conducted in the Qilian Mountain of the northeastern QTP that examined a wet alpine pasture dominated by *K. humilis* subject to moderate sheep grazing, suggested that aboveground net primary production (ANPP) increased slightly (relative to an un-grazed control) in two of studied 5 years (Wang et al., 2012). Yak grazing in degraded sedge meadows on the southern Qinghai Province have resulted in consistent overcompensatory growth during short-time treatments, albeit with various intensities (Dong et al., 2012). In contrast, sheep grazing experiments in the dry grasslands of Tibet reduced the ANPP in direct proportion to increases in stocking rates (Ganjurjav et al., 2015). A detailed evaluation based on more extensive manipulative experiments is therefore needed to clarify the effect of grazing on plant production and test the grazing optimization hypothesis in the QTP alpine pastures.

Unlike sheep, horses, or other livestock, which were introduced to the QTP, yaks have been domesticated and raised as a native livestock in this region for over 7000 years (Wang et al., 2010; Mipam et al., 2012; Qiu et al., 2015). Therefore, the alpine pasture may have initially been established via co-evolution with grazing herds of yaks. In the present study, we tested the grazing optimization hypothesis in a QTP pasture. Grazing experiments were conducted in a degraded pasture dominated by *K. pygmaea*, located in Hongyuan County in the eastern QTP. Local investigations and Tibetan Buddhist records indicate that this area has been continuously grazed by yaks for several centuries. Specifically, we hypothesized that (i) yak grazing might lead to plant compensation (especially moderate grazing intensity) in alpine pasture habitats and (ii) the level of plant compensation might change with grazing intensity and time.

## Materials and Methods

### Study Site

**FIGURE 1:**
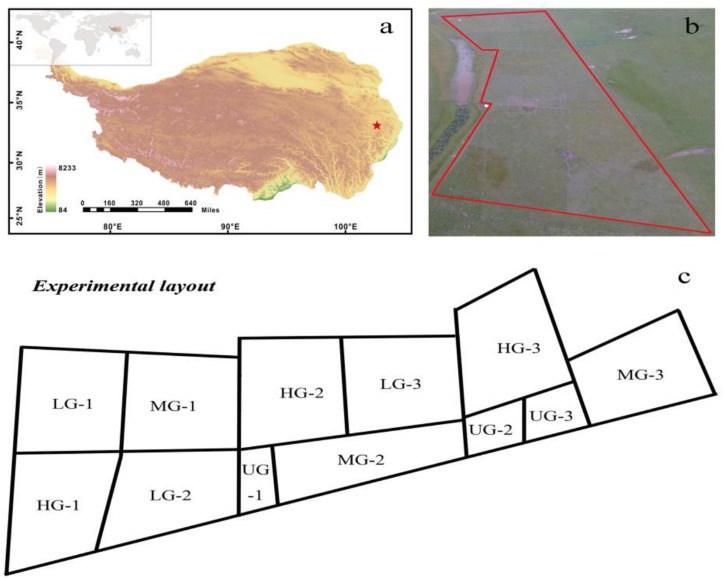
Location (red star, **a**), aeroview **(b)** and experimental layout **(c)** of the yak-grazing experiment in the eastern Qinghai-Tibet Plateau. Each grazing intensity consists of three replicates (e.g., 1, 2, and 3). The main plots were divided into four grazing intensities. UG, un-grazed; LG, light grazing; MG, moderate grazing; HG, heavy grazing.

**FIGURE 2:**
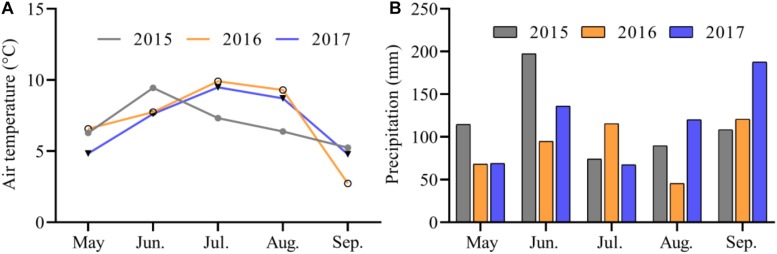
Monthly mean air temperature **(A)** and precipitation **(B)** at the experimental site during the growing seasons from 2015 to 2017.

The study site is a typical alpine pasture located at the Qinghai-Tibetan Plateau Research Base of Southwest Minzu University (32°48′N, 102°33′E), which is 5 km from Hongyuan County, Sichuan Province, China (Figure 1). The site’s mean elevation is around 3504 m, and its mean annual temperature between 1961 and 2013 is 1.5°C, with an average temperature of 11.1°C in the warmest month (July) and –9.7°C in the coldest month (January). The mean annual precipitation is about 747 mm, with 80% falling from May to September. The monthly mean air temperature and precipitation during the growing seasons (May–September) varied over the 3 years (2015–2017) of the experimental period (Figure 2). The vegetation at the experimental site was dominated by *K. pygmaea*. Other species present included several *Kobresia* and *Carex* species; grass (Poaceae) species (*Elymus nutans*, *Deschampsia caespitosa*, *Poa annua*, and *Agrostis* spp.); and forb species (*Ligularia virgaurea* (*Asteraceae*), *Anemone rivularis* (*Ranunculaceae*), *Saussurea* and *Leontopodium* (*Asteraceae*) spp., and *Potentilla* spp. (*Rosaceae*). The most common small mammals at the site were zokors, with a small quantity.

### Experimental Design

Previous grazing studies in the QTP defined the light, moderate and heavy grazing intensities as 0.89, 1.45 and 2 yak units per hectare, respectively (Dong et al., 2012). However, grazing intensity may differ in various regions. Our investigations and discussions with local herdsmen indicated that the typical yak grazing intensity in this region was three or more yak units per hectare. We therefore set the heavy grazing intensity at this level in the experiments. The experimental site was established in a 10-ha flat meadow pasture and the grazing experiment started in late May in 2015. We divided the experimental site into 12 plots by erecting fences (Figure 1). Three plots were subject to light yak grazing (LG; one yak unit per hectare), three to moderate grazing (MG; 2 yak units per hectare), and three to heavy grazing (HG; 3 yak units per hectare). The remaining plots were un-grazed (UG) controls. There were thus three replicates plots for each treatment. All plots were distributed within the site over an area of 10 ha (Figure 1). No neighboring plots shared a common treatment other than the UG-2 and UG-3 plots. Each grazed plot covered 1 ha, and the three un-grazed plots collectively covered an area of 1 ha. The body weights of yaks were approximately 200 kg. In each of the studied 3 years, the grazing experiments were initiated in late May and terminated in late September; for the remaining months of each year, the experimental plots were left un-grazed to simulate summer meadow pastures.

### Estimation of ANPP and Plant Diversity

The ANPP in the UG plots was determined by collecting total aboveground live plant biomass at each month. Biomass was collected from three 50 cm × 50 cm quadrats to minimize the effect of heterogeneity. The ANPP for the grazed plots was estimated using the temporary cage method (McNaughton et al., 1996; Knapp et al., 2012). Briefly, for each grazing plot, we used four (1 m × 1 m × 1 m) temporary cages and paired grazing subplots to examine ANPP at monthly intervals during the experimental period (from late-May to late-September for each year). We moved the temporary cages and established new paired grazing subplots at monthly intervals during the experimental period to minimize error related to frequency sampling (Knapp et al., 2012). We estimated the ANPP in each grazed plot by collecting aboveground live plant biomass from a 50 cm × 50 cm quadrat within each temporary cage and from the paired grazing subplot before moving them to the new subplot. In total, we collected biomass samples from 675 quadrats to examine the response of ANPP to grazing intensity from 2015 to 2017. All aboveground live plant biomass was collected at ground level and then dried at 65°C for 48 h to a constant weight. For all plots, litter and dead biomass from previous years was excluded when computing ANPP to obtain accurate estimates of plant growth in each sampling interval (Knapp et al., 2012). To compute the ANPP for each grazed plot, we first subtracted the biomass of the paired grazed subplot from that inside the temporary cage at each sampling interval. We then summed the differences during the experimental period for each year. The sum of the accumulative positive differences and the remaining biomass in the paired grazed subplots at the September was recorded as the ANPP. In the first interval of the growing season, we assumed there was no significant difference in biomass between the temporary cage and the paired grazed subplot (Knapp et al., 2012). It is worth noting that the moveable-cage method outperforms alternative methods for detecting compensatory growth despite its inherent uncertainties (McNaughton et al., 1996). In addition, to examine the effect of grazing intensity on community compositions, we also recorded the biomass of individual functional groups (*Poaceae, Cyperaceae, Asteraceae, Fabaceae* and “*Others*”) at the end of the growing season (September).

Plant species coverage and frequency were determined by visual estimation for each plant species (Supplementary Tables S1, S2). Plant diversity including species richness and Shannon–Wiener diversity index were evaluated. The Pielou’s evenness index was also calculated for each plot because it reflects relative species abundance. These indices have been used extensively in diverse ecosystems (e.g., Tóth et al., 2018). Species richness was determined by counting the number of plant species in each investigated quadrat. The Shannon–Wiener diversity index and Pielou’s evenness index were calculated based on the relative importance and abundance of plant species, using the following equations:

$$H^{\prime }=-\sum_{i=1}^{s}P\,i\,L\,n\,P\,i$$

$$\;J=H^{\prime }/L\,n\,S$$

Where *H*′ is the Shannon–Wiener diversity index, *J* is the Pielou’s evenness index, *S* is species richness, and *Pi* represents the relative importance of species *i* in each quadrat, which is calculated as the ratio of the abundance of species *i* to the total abundance of all species.

### Statistical Analyses

The normality and homogeneity of variance of each dataset were tested using the Shapiro–Wilk method. We used two-way Generalized Linear Models (GLMs) to examine the differences in plant diversity, ANPP and the plant biomass of individual functional groups between grazing intensities and years, in which individual measurements were nested in plots and grazing intensity and year were treated as fixed factors. The datasets were normalized by log-transformation where necessary to enable robust analysis before conducting analysis of variance (ANOVA). A *post hoc* test (least significant difference, LSD) was then performed to test the significance of differences between grazing treatments and years. These calculations were performed using SPSS Statistics version 21 (SPSS Inc., Chicago, IL) with a significance threshold of *P* < 0.05.

## Results

### Plant Diversity and Coverage

**FIGURE 3:**
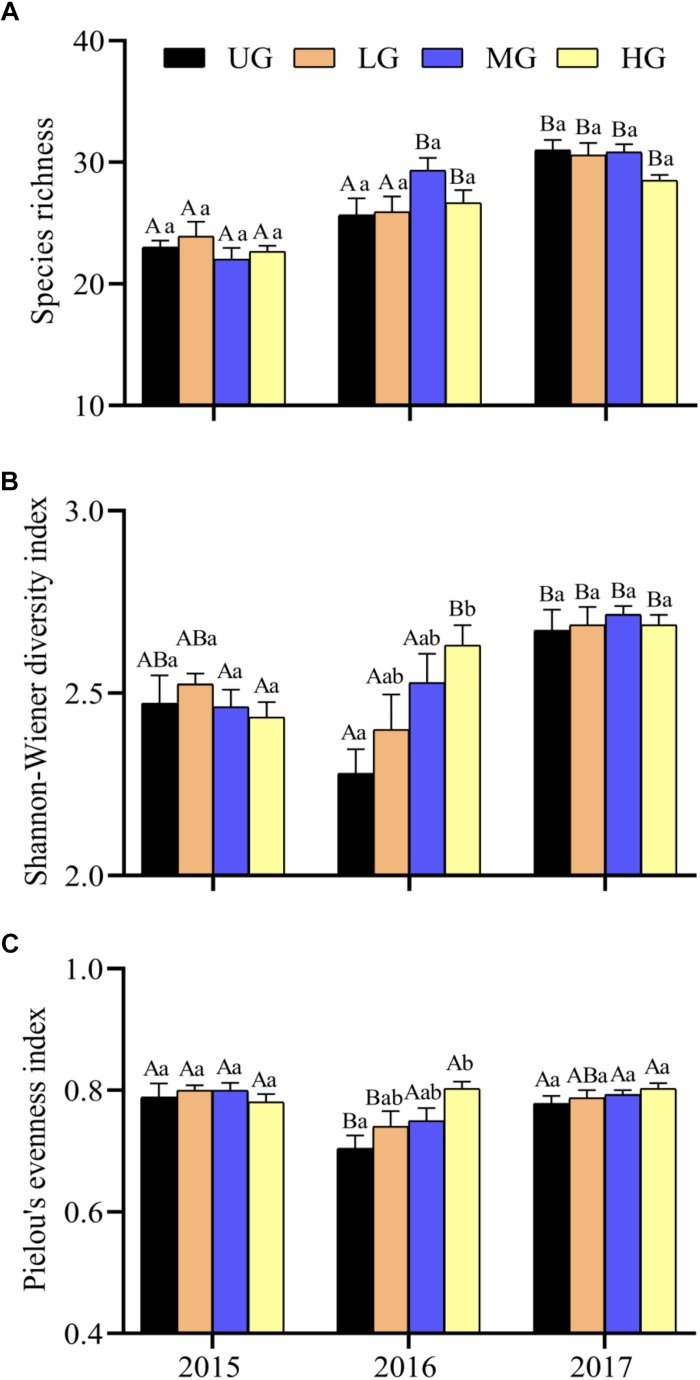
Plant species richness **(A)**, Shannon–Wiener diversity index **(B)** and Pielou’s evenness index **(C)** under different grazing intensities from 2015 to 2017. Bars indicate standard errors. Different lowercase letters (a,b) above the bars indicate significant differences between grazing intensities for each year. Significant differences between different years within same grazing intensity are shown by capital letters (A,B). UG, un-grazed; LG, light grazing; MG, moderate grazing; HG, heavy grazing.

Species richness, Shannon–Wiener diversity index and Pielou’s evenness index varied significantly between years and grazing treatments (Figure 3 and Table 1). Species richness increased significantly from 2015 to 2017 under all treatments but did not differ significantly between grazing treatments in any year. The Shannon–Wiener diversity index initially decreased and then increased under UG and LG, whereas it gradually increased under MG and HG from 2015 to 2017. There were no significant differences between UG and grazing treatments in 2015 and 2017, but the index was significantly lower in UG plots than that in HG plots in 2016 (Figure 3). Pielou’s evenness index decreased from 2015 to 2016 but increased from 2016 to 2017 under the UG, LG and MG treatments. Similar to Shannon–Wiener diversity index, no significant differences were observed for Pielou’s evenness index among grazing treatments in 2015 and 2017 (Figure 3). GLMs analyses showed that grazing intensity marginally affected Pielou’s evenness index but not species richness or Shannon–Wiener diversity index, whereas year exerted significant effects on all indices (Figure 3 and Table 1). The interaction of grazing intensity and year did not affect species richness and Pielou’s evenness index, but did significantly affect Shannon–Wiener diversity index (Table 1). These results suggest that year, rather than grazing intensity, significantly influenced plant diversity during the 3 years of the yak grazing experiment. On average, species richness, Shannon–Wiener diversity index, and Pielou’s evenness index ranged from 25.9 to 27.4, 2.50 to 2.58, and 0.76 to 0.80, respectively, among grazing treatments from 2015 to 2017 (Table 2).

**TABLE 1:** Generalized linear models showing the differences in plant diversity, ANPP and the plant biomass of individual functional groups between grazing intensities and years.

**Factors**	**ANPP**		**SR**		**SWD**		**PE**		***Poaceae***		***Cyperaceae***		***Fabaceae***		***Asteraceae***		***Others***	
									
	***F***	***P***	***F***	***P***	***F***	***P***	***F***	***P***	***F***	***P***	***F***	***P***	***F***	***P***	***F***	***P***	***F***	***P***
GI	15.53	< 0.001	0.78	0.508	1.76	0.158	2.70	0.049	15.14	< 0.001	14.81	< 0.001	4.64	0.004	4.85	0.003	12.05	< 0.001
Y	11.95	< 0.001	54.93	< 0.001	18.6	< 0.001	9.30	< 0.001	9.65	< 0.001	9.92	< 0.001	2.64	0.076	3.81	0.025	12.69	< 0.001
GI × Y	2.35	0.035	1.89	0.088	2.41	0.031	2.12	0.055	0.82	0.56	1.03	0.41	0.66	0.68	2.27	0.041	1.24	0.29

*ANPP, aboveground net primary production; GI, grazing intensity; Y, year; SR, species richness; SWD, Shannon–Wiener diversity index; PE, Pielou’s evenness index.*

**TABLE 2:** Changes in ANPP, plant diversity and the plant biomass of functional groups for each grazing intensity during the growing seasons from 2015 to 2017 (mean ± standard error).

	**Grazing intensities**			
	
	**UG**	**LG**	**MG**	**HG**
ANPP (g/m^2^)	334.13±13.87^a^	435.97±15.93*bc*	475.20±17.83^c^	396.77±19.38^b^
Species richness	26.67±0.88^a^	26.81±0.80^a^	27.39±0.82^a^	25.94±0.57^a^
Shannon–Wiener diversity index	2.50±0.05^a^	2.54±0.04^a^	2.57±0.04^a^	2.58±0.03^a^
Pielou’s evenness index	0.76±0.01^a^	0.78±0.01^a^	0.78±0.01^a^	0.80±0.01^a^
Biomass of *Poaceae* (g/m^2^)	87.16±14.48^a^	38.86±5.69^b^	20.91±3.29^c^	14.67±1.96^c^
Biomass of *Cyperaceae* (g/m^2^)	60.02±6.80^a^	52.09±9.98^a^	27.12±3.06^b^	17.76±1.88^b^
biomass of *Fabaceae* (g/m^2^)	15.34±2.70^a^	8.39±1.38^b^	9.33±1.42*ab*	6.51±0.86^b^
Biomass of *Asteraceae* (g/m^2^)	62.69±9.06*ab*	77.03±9.79^a^	47.78±6.29*ab*	36.13±3.80^b^
Biomass of *Others* (g/m^2^)	95.85±5.34^a^	60.04±4.58^b^	65.03±6.38^b^	51.97±4.29^b^

*ANPP, aboveground net primary production; UG, un-grazed; LG, light grazing; MG, moderate grazing; HG, heavy grazing. Different lowercase letters indicate significant differences between grazing intensities.*

Species coverage and frequency for individual plant species also varied substantially between grazing treatments and years. For example, the coverage of *P. annua* and *K. humilis* decreased gradually from 2015 to 2017 under all grazing treatments. In contrast, the coverage of *K. pygmaea* increased and while that of *Ajania potaninii* was relatively stable (Supplementary Tables S1, S2).

### ANPP and Standing Biomass of Plant Functional Groups

**FIGURE 4:**
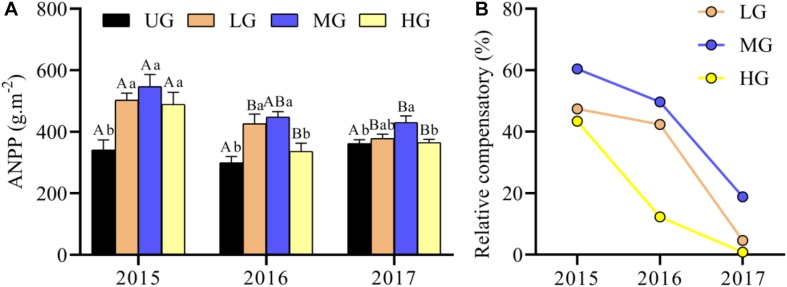
Response of ANPP **(A)** and relative compensatory **(B)** to yak grazing intensity from 2015 to 2017. Bars indicate standard errors. Relative compensatory is calculated as the differences between each grazing treatment relative to un-grazing plots. Different lowercase letters (a,b) above the bars indicate significant difference between grazing treatments each year. Significant differences between different years within a grazing treatment are indicated by capital letters (A,B). ANPP, aboveground net primary production. UG, un-grazed; LG, light grazing; MG, moderate grazing; HG, heavy grazing.

The ANPP under grazing treatments (LG, MG and HG) was generally higher compared with that under UG treatment (Figure 4A). Commonly, the ANPP was highest in MG plots, followed by LG, HG and UG, with a significant difference between UG and MG (Figure 4A). There were no significant differences in ANPP between years under UG, but the ANPP under grazing treatments was significantly higher in 2015 than in 2017 (Figure 4A). GLMs analyses suggested that ANPP was significantly affected by grazing intensity, year, and their interaction (Table 1). The mean ANPP over the experimental period was 334.13, 435.97, 475.20, and 396.77 g/m^2^ for the UG, LG, MG, and HG treatments, respectively (Table 2). Moreover, the relative compensatory decreased dramatically under grazing treatments from 2015 to 2017 (Figure 4B). The relative compensatory under MG and LG decreased slightly from 2015 (MG, 60.4%; LG, 47.5%) to 2016 (MG, 49.7%; LG, 42.4%) and then sharply decreased to 2017 (MG, 18.8%; LG, 4.6%), while it drastically declined under HG plots from 43.4% in 2015 to 12.3% in 2016 and then to 0.8% in 2017. This implied that the grazing-induced ANPP improvement may last only several years.

**FIGURE 5:**
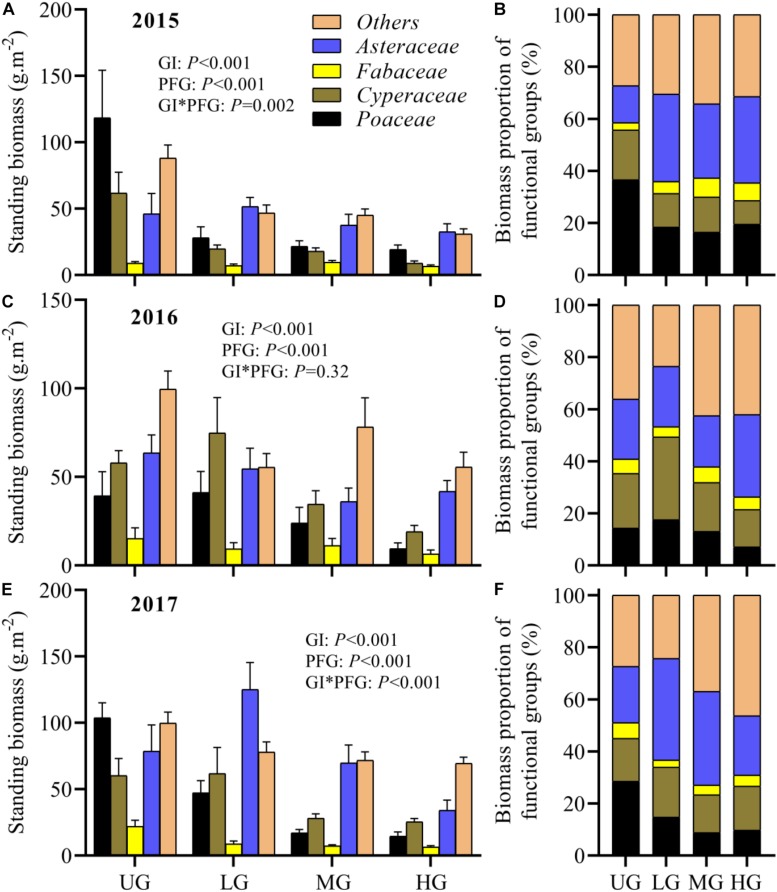
Plant standing biomass and corresponding proportion of each functional group among different grazing treatments and years (2015: **A,B**; 2016: **C,D**; 2017: **E,F**). Bars indicate standard errors. GI, grazing intensity; PFG, plant functional group; UG, un-grazed; LG, light grazing; MG, moderate grazing; HG, heavy grazing.

Grazing intensity significantly affected the standing biomass of individual functional groups, and its effect changed gradually over time. In 2015, the standing biomass of *Poaceae*, *Cyperaceae* and *others* was significantly higher in UG plots than in grazed plots, while the standing biomass of *Fabaceae* and *Asteraceae* exhibited no significant differences among grazing treatments (Figures 5A,B). In 2016, the standing biomass of *Poaceae*, *Fabaceae*, *Asteraceae* and *others* showed no significant differences among grazing treatments, though their values varied substantially (Figures 5C,D). The standing biomass of *Cyperaceae* under LG was significant higher compared with that under MG and HG (Figures 5C,D). However, in 2017, the standing biomass of *Asteraceae* was much higher in LG plots, and *Fabaceae* and *Poaceae* exhibited higher standing biomass in UG plots (Figures 5E,F). The *Cyperaceae* biomass under UG and LG exceeded that under MG and HG, but there were no significant differences in the biomass of *others* species among grazing treatments (Figures 5E,F).

The standing biomass of individual functional groups also varied significantly with time. For instance, the biomass of *Fabaceae* in UG plots increased gradually during the experimental period but did not change significantly under the grazing treatments (Figure 5). In contrast, the biomass of *others* did not vary significantly in UG plots from 2015 to 2017 but increased in grazed plots (Figure 5). Moreover, GLMs analyses indicated that both grazing intensity and year significantly affected the plant biomass of all functional groups except *Fabaceae*, which was not influenced by year (Table 1). The interaction of grazing intensity and year had no effect on plant biomass for any functional group, with an exception of *Asteraceae* (Table 1). On average, the standing biomass of each functional group decreased significantly as grazing intensity increased (Table 2).

## Discussion

### Species Diversity and Coverage in Response to Yak Grazing

Plant composition is known to positively affect ANPP in grassland ecosystems (Lezama et al., 2014). Previous studies indicated that low or moderate grazing is likely to increase plant diversity by shifting biotic factors (Borer et al., 2014; Eldridge et al., 2016). A recent study has also reported that cattle grazing had little effect on plant diversity in a high species diversity grassland during a 2-year grazing experiment (Liu et al., 2015). However, some studies have found that grazing does significantly impact plant diversity. For example, in South American grazing grassland ecosystems, species richness decreased by 36% in the least productive regions but increased by 106% in the most productive regions (Lezama et al., 2014).

It has been suggested that low and moderate levels of sheep grazing increase species diversity in QTP alpine pastures (Ganjurjav et al., 2015). However, species richness varied significantly between years in our experimental plots without exhibiting consistent changes in response to yak grazing intensity (Figure 3 and Table 1). Our result indicated that plant diversity in the third year exceeded that during the first year under all grazing regimes. This difference between our results and previous studies can be attributed to two reasons. The first is that differences in foraging patterns, intensities and species preferences between Tibetan sheep and yaks may lead to contrasting vegetation composition (Eldridge et al., 2016). Sheep can select plants by dexterous grazing, while yaks consume mixed plants without careful selection despite favoring grasses and sedges (Squires, 1981; Letnic, 2004). Therefore, grazing by sheep and yaks can affect vegetation composition differently due to dissimilarities in their consumption of particular plant species. Second, plant community composition may be insensitive to short-term changes in grazing intensity in ecosystems with a long history of grazing (Milchunas et al., 1988). The alpine pasture dominated by *Kobresia* species in the QTP may have been artificially controlled by yak grazing for several thousand years (Miehe et al., 2019). Most regions may have experienced similar levels of over-grazing for an equally long time. Thus, short-term variation in grazing intensity may not substantially affect species diversity. However, species diversity increased in all plots over successive years. Further long-term monitoring of species composition based on *in situ* grazing experiments is needed to clarify the complex relationships between herbivores and plant diversity across the alpine pastures.

### Plant Compensation in Response to Yak Grazing

Our results provide experimental evidence that ANPP is strongly affected by yak grazing intensity. In particular, moderate grazing greatly enhanced the ANPP in all grazed plots during the experimental period. This is essentially consistent with the results of earlier grazing intensity experiments in Qinghai Province (Dong et al., 2012), North America (Hik and Jefferies, 1990; Alward and Joern, 1993), Africa (McNaughton, 1989; Leriche et al., 2003; Charles et al., 2017) and Inner Mongolia (Liu et al., 2011), confirming that plants can undergo compensatory growth when grazed by large herbivores. This overcompensation also clearly occurred in 2016 under light grazing (Figure 4). Together with the results of previous yak grazing experiments in QTP alpine meadows dominated by *Kobresia* species (Dong et al., 2012), our results suggest that the level of plant overcompensation varies over time (i.e., between successive grazing experiments). While the level of overcompensation decreased continuously over time, it remained higher under moderate grazing than under other grazing regimes (Figure 4). However, our results are inconsistent with another study in which no grazing-induced overcompensation was observed in grassland in the Tibet Autonomous Region (Ganjurjav et al., 2015). This difference is probably due to the large differences in mean annual precipitation among sites (348 mm at the Tibetan site, 476 mm at the Qinghai site and 747 mm at our site). Lu et al. (2017) suggested that precipitation of less than 400 mm during the growing season could limit the ANPP in the QTP alpine ecosystems. Thus, plant compensation induced by grazing may occur in regions where annual precipitation exceeds 400 mm. Two other empirical studies have also revealed that grazing effects on plant production, abundance and richness can vary greatly with precipitation gradients (Socher et al., 2013; Eldridge et al., 2016). In fact, there are many factors that could contribute to variation in ANPP (and thus plant overcompensation), such as the photosynthetic rates of residual tissue, light availability, and nutrient cycling of dung and urine (McNaughton, 1989; Borer et al., 2014). Moreover, the relative compensatory effect gradually decreased during the experimental period, with no apparent compensatory effect at LG and HG plots after 3 years of grazing treatments (Figure 4B), suggesting that grazing-induced plant overcompensation occurs in the short term but not in the long term. It has been reported that vegetative component may overgrow under proper herbivory pressure due to the original phenology, leading to excess vegetative biomass beyond the optimal size of vegetative parts and thus short-term grazing optimization (Hayashi et al., 2007).

There are several major patterns associated with plant overcompensation. First, the excessive feeding by herbivores on dominant vegetation species may impair the light competition of those species, promoting the growth of plants with limited light access and thus enhancing plant production (Borer et al., 2014). In this study, only minor differences in the standing biomass of *Fabaceae*, *Asteraceae* and *Others* were observed between grazing treatments and years (Figure 5). It may therefore be that there was appreciable light competition between plant functional groups during the experimental period. Second, the feeding of herbivorous livestock on their favorite plants can improve plant production through promoting apical dominance and photosynthetic capacity and reducing plant fitness (Rautio et al., 2005; Zhang et al., 2015). Our results indicated that the standing biomass of *Poaceae* and *Cyperaceae* in MG plots was much lower than that in UG plots (Figure 5). Together with the higher ANPP in MG plots, this may indicate that the increased ANPP under moderate grazing intensity was partly due to the stimulation of *Poaceae* and *Cyperaceae* growth. However, the low standing biomass of these two groups in HG plots suggests that the insignificant plant overcompensation under heavy grazing intensity may not be attributed to variation in the growth of *Poaceae* and *Cyperaceae* (Figure 5). Thus, more evidence is needed to properly test these hypotheses. Moreover, resource use efficiency and photosynthetic rates affect plant overcompensation. Accelerated or regulated nutrient cycling from the herbivore’s excretory substances may stimulate plant regrowth (Hayashi et al., 2007). The improvement of photosynthetic rate after grazing produces more non-photosynthetic tissue than young leaves (Hayashi et al., 2007).

Compensatory growth not only has an effect on ANPP but also on belowground net primary production (BNPP) due to plant biomass allocation. It has been reported that heavy grazing significantly declined BNPP due to carbon reallocation to shoot growth in Inner Mongolia (Gao et al., 2008). In a temperate grassland of the South America, grazing showed a higher BNPP compared to ungrazed regions (López-Mársico et al., 2015). Moreover, no responses of BNPP to grazing were obtained in both the northern Tibetan Plateau and Inner Mongolia grasslands (Gong et al., 2015; Zeng et al., 2015), but grazing decreased the proportion of BNPP to NPP and thus grazing optimization on ANPP at the expense of BNPP should potentially reduce soil quality (Gong et al., 2015). However, the belowground data were not available in the present study and, thereby, further works should focus on the response of belowground processes to grazing intensity in alpine pastures.

Although this study focused on plant compensation (which can only occur during growing seasons), there may be differences in compensation between grassland pastures (e.g., winter pasture and summer pasture) due to different grazing practices. It has been reported that grazing had a greater negative impact on preferred plants in winter pastures than in summer pastures because of differences in stocking rates in the Tibetan grassland (Dong et al., 2015; Harris et al., 2016). Nevertheless, the underlying mechanisms responsible for grassland degradation in different pastures remain unclear (Harris et al., 2015), and will need to be clarified by further *in situ* experiments.

### Factors Affecting ANPP Under Grazing Intensities

During the experimental period, resource availability was possibly the main contributor to variations in ANPP under different grazing intensities, since some factors can be excluded such as fire history and co-evolutionary history that were not significantly affected by grazing intensity in this study. It has been reported that the dominant factors affecting resource availability in grassland ecosystems are precipitation, air temperature, nutrient availability, and soil water content (Frank et al., 2018). Previous studies have indicated that precipitation has a positive (Lezama et al., 2014; Lu et al., 2017) or neutral (Wang et al., 2012; Eldridge et al., 2016) effect on primary production in grasslands on a regional scale; in contrast, the timing of rainfall events and precipitation periods are key factors to affect ANPP on a plot scale (Knapp et al., 2012). Similarly, air temperature has a positive effect on ANPP (Wang et al., 2012; Lu et al., 2017). However, precipitation and air temperature were not the main factors to lead to the variations in ANPP under different grazing intensities due to the relative narrow area in the present study. Nutrient availability is a key factor limiting ANPP in terrestrial ecosystems and plays a dominant role in controlling ANPP at smaller spatial scales (La Pierre et al., 2016). Grazing can promote the exudation of nutrients from roots and enhance urine and dung released by herbivores, increasing nutrient availability and thus ANPP in alpine pastures (Lu et al., 2017). Soil moisture also plays a critical role in promoting plant growth, especially in Tibetan grasslands (Zhao et al., 2011; Zhang et al., 2015). Anderson et al. (2007) suggested that soil moisture strongly affected grazing overcompensation when other factors remained stable. Furthermore, previous studies indicated that nitrogen and water additions can enhanced ANPP in grazed area with different intensities in Inner Mongolia grasslands, with decreasing responses with increasing grazing intensities (Li et al., 2011; Gong et al., 2015). La Pierre et al. (2016) also found that the interaction of nutrient and water use efficiency mainly controlled ANPP at a site level. Thus, it is reasonable to infer that nutrient availability and soil moisture may be key factors that affect ANPP under different grazing intensities in this specific ecosystem. However, further long-term experiments with continuous observation are required to elucidate the effect of yak-grazing on the interaction between resource availability and plant production.

## Conclusion

In summary, our results have experimentally indicated that yak grazing can increase ANPP in alpine meadows of the eastern QTP, especially when the grazing intensity is moderate, suggesting that grazing optimization occurs in the short term but not in the long term. The degree of plant overcompensation under moderately intense grazing exceeded that under heavy grazing, possibly because of the regrowth of specific functional groups in the plant community composition (mainly *Poaceae* and *Cyperaceae*). Our study will support the development of an ecological conservation strategy that will help restore the fragile ecosystems of the QTP and balance the seemingly incompatible requirements of animal husbandry in QTP alpine meadows and their ecological conservation.

## Data Availability

All datasets generated for this study are included in the manuscript and/or the Supplementary Files.

## Author Contributions

T-DM and J-QL conceived and designed the study. T-DM, L-LZ, and GM collected the data. T-DM, L-LZ, and L-MT analyzed the data. T-DM and L-MT led the writing of the manuscript. All authors contributed critically to the manuscript and gave final approval for publication.

## Conflict of Interest Statement

The authors declare that the research was conducted in the absence of any commercial or financial relationships that could be construed as a potential conflict of interest.
